# Editorial: Microbial food safety in retail stores and restaurants

**DOI:** 10.3389/fmicb.2024.1518203

**Published:** 2024-11-19

**Authors:** Zahra H. Mohammad, Faizan Ahmad

**Affiliations:** ^1^Conrad N. Hilton College of Hotel and Restaurant Management, University of Houston, Houston, TX, United States; ^2^Post Harvest Engineering and Technology, Faculty of Agricultural Sciences, Aligarh Muslim University, Aligarh, UP, India

**Keywords:** food services, bacterial contamination, public health, food safety, GMP

Microbial contamination is a significant concern for food safety and public health, contributing to a high incidence of global foodborne illnesses. It has the potential to occur at any point along the food production chain (Tropea, 2022).

Despite advancements in food safety measures and practices, more than half of foodborne illness outbreaks in the U.S. are still associated with restaurants and food service businesses (DeWaal and Glassman, 2013). This poses significant public health risks and exposes those businesses accountable for the outbreaks to potential legal, financial, and reputational damage (Food Safety Focus National Restaurant Association, 2004).

Unfortunately, in our daily interactions with food retail stores and food service establishments, whether browsing the aisles at the grocery store, dining out at a restaurant, or ordering takeout, we naturally assume that the food we purchase is safe for consumption. The food supply chain works tirelessly to sustain/meet this expectation; however, food safety issues continue to arise, leading to unsafe food and the need for food recalls (Nestle, 2010). While these problems commonly originate from the earlier stages of the supply chain, cases of contamination occur within retail establishments—both in grocery stores and food service settings (Linton, 1996; Lianou and Sofos, 2007; Moritz et al., 2023).

Furthermore, under this Research Topic, several scientific research studies have presented the findings regarding the prevalence and antibiotic resistance profile of bacteria, such as Staphylococcus spp, Listeria spp, E. coli, Salmonella, *Vibrio alginolyticus* and contamination of *Vibrio parahaemolyticus* in crayfish at food services and retail settings settings (Angamarca et al.; Punchihewage-Don et al.; Wu et al.; Sun et al.). Additionally, an aflatoxin B_1_-degrading strain from the Qinghai-Tibet Plateau was presented as potential microbial contamination (mold) at food services operations (Tang et al.). The primary aim of this Research Topic was to demonstrate how the microbial risk at retail and food service operations poses a public health concern and to increase knowledge about potential microbial contamination in these settings.

The prevalence of microbial contamination in retail and food service operations is due to a lack of a food safety management system, resulting in poor personal hygiene and not following good manufacturing practices (Moritz et al., 2023). Insufficient training and food safety knowledge of food handlers and other staff members and poor attitudes toward the seriousness of food safety could lead to practices that increase the risk of food contamination (Johnson et al., 2004).

Understanding the prevalence of microbes and antibiotic resistance is essential for adapting and promoting a food safety culture, implementing the best food safety practices, and ensuring adherence to personal hygiene standards in retail and food service establishments.

Therefore, this Research Topic presents the serious issue of microbial contamination in retail stores and food service establishments. It highlights that pathogenic microorganisms could contaminate food products at any point in the supply chain or production process, posing a consequential food safety risk to public health due to potential foodborne illnesses.

To conclude, good manufacturing practices (GMP), good personal hygiene, regular employee training, and food safety management are essential to prevent microbial contamination and mitigate foodborne illnesses at retail and food service establishments.

## References

[B1] DeWaal C. S. Glassman M. (2013). Outbreak Alert! 2001–2010: Center for Science in the Public Interest. Available at: https://www.cspinet.org/sites/default/files/media/documents/resource/outbreakalert2014.pdf (accessed October 18, 2024).

[B2] Food Safety Focus National Restaurant Association (2004). Available at: https://foodsafetyfocus.com/blog/october-2024/nfsm-2024-week-6 (accessed October 18, 2024).

[B3] Johnson L. Shin J. H. Feinstein A. H. Mayer K. J. (2004). Validating a food safety instrument: measuring food safety knowledge and attitudes of restaurant employees. J. Foodserv. Busin. Res. 6, 49–65. 10.1300/J369v06n02_05

[B4] Lianou A. Sofos J. N. (2007). A review of the incidence and transmission of Listeria monocytogenes in ready-to-eat products in retail and food service environments. J. Food Protect. 70, 2172–2198. 10.4315/0362-028X-70.9.2172 17900099

[B5] Linton R. (1996). Food Safety Hazards in Foodservice and Food Retail Establishments. West Lafayette, IN: Department of Food Science, Purdue Cooperative Extension Service.

[B6] Moritz E. D. Ebrahim-Zadeh S. D. Wittry B. Holst M. M. Daise B. Zern A.. (2023). Foodborne illness outbreaks at retail food establishments - National Environmental Assessment Reporting System, 25 state and local health departments, 2017–2019. MMWR Surveill Summ. 72, 1–11. 10.15585/mmwr.ss7206a1 37252900 PMC10231936

[B7] Nestle M. (2010). Safe food: The Politics of Food Safety (Vol. 5). California: University of California Press.

[B8] Tropea A. (2022). Microbial contamination and public health: an overview. Int. J. Environm. Res. Public Health 19:7441. 10.3390/ijerph19127441 35742689 PMC9224327

